# Supplementary material for: Immune dynamics in SARS-CoV-2 experienced immunosuppressed rheumatoid arthritis or multiple sclerosis patients vaccinated with mRNA-1273
Source: eLife. 2022 Jul 15;11:e77969. doi: 10.7554/eLife.77969 (PMC9337853; doi:10.7554/eLife.77969)
Supplement: Supplementary file 1. — Table showing characteristics of participants divided in to immune-mediated inflammatory disorder (IMID) patients with immunosuppressants and healthy controls. [file elife-77969-supp1.docx]

|  |  | All (n=28) | Relapsing Remitting MS  (n=7) | Rheumatoid Arthritis  (n=6) | Healthy controls  (n=15) |  |
| --- | --- | --- | --- | --- | --- | --- |
|  | **Age, in years, median (range)** | 49.5 (23-61) | 50 (37-53) | 47.5 (40-61) | 49 (23-57) |  |
|  | **Sex** |  |  |  |  |  |
|  | Female | 14 (50) | 3 (43) | 3 (50) | 8 (53) |  |
|  | Male | 14 (50) | 4 (57) | 3 (50) | 7 (47) |  |
|  | **Comorbidities** |  |  |  |  |  |
|  | Diabetes | 0 | 0 | 0 | 0 |  |
|  | Obesity | 2 (7) | 0 | 2 (33) | 0 |  |
|  | Hypertension | 0 | 0 | 0 | 0 |  |
|  | Cardiovascular disease | 1 (4) | 0 | 0 | 1 (7) |  |
|  | Pulmonary disease | 0 | 0 | 0 | 0 |  |
|  | Hematological malignancy or chemotherapy in past 2 years | 0 | 0 | 0 | 0 |  |
|  | Chronic kidney disease | 0 | 0 | 0 | 0 |  |
|  | Other autoimmune disease | 2 (7) | 0 | 2 (33) | 0 |  |
|  | **Time between SARS-CoV-2 infection and vaccination** |  |  |  |  |  |
|  | 56-180 days | 12 (43) | 4 (57) | 2 (33) | 6 (40) |  |
|  | >180 days | 16 (57) | 3 (43) | 4 (67) | 9 (60) |  |
|  | **WHO COVID-19 case definition (1)** |  |  |  |  |  |
|  | Confirmed | 28 (100) | 7 (100) | 6 (100) | 15 (100) |  |
|  | Probable | 0 | 0 | 0 | 0 |  |
|  | Suspected | 0 | 0 | 0 | 0 |  |
|  | **COVID-19 severity of infection*** |  |  |  |  |  |
|  | Asymptomatic | 2 (7) | 0 | 0 | 2 (13) |  |
|  | Mild | 24 (86) | 6 (86) | 5 (83) | 13 (87) |  |
|  | Moderate | 2 (7) | 1 (14) | 1 (17) | 0 |  |
|  | Severe | 0 | 0 | 0 | 0 |  |
|  | **Number of mRNA-1273 vaccinations** |  |  |  |  |  |
|  | One | 28 (100) | 7 (100) | 6 (100) | 15 (100) |  |
|  | Two | 24 (86) | 6 (86) | 6 (100) | 12 (80) |  |
|  | **Immunosuppressive therapy at time of primo infection** |  |  |  |  |  |
|  | Ocrelizumab | 6 (21) | 6 (86) | 0 | 0 |  |
|  | Cumulative infusions at time of infection, median(range) | 4 (0-5) | 4 (1-5) | 0 | 0 |  |
|  | Ocrelizumab <3 months from last infusion to infection | 1 (4) | 1 (14) | 0 | 0 |  |
|  | Ocrelizumab >3 months from last infusion to infection | 5 (18) | 5 (72) | 0 | 0 |  |
|  | Methotrexate** | 6 (21) | 0 | 6 (100) | 0 |  |
|  | None | 16 (57) | 1 (14) | 0 | 15 (100) |  |
|  | **Immunosuppressive therapy at time of vaccination** |  |  |  |  |  |
|  | Ocrelizumab | 7 (25) | 7 (100) | 0 | 0 |  |
|  | Cumulative infusions at time of vaccination, median (range) | 5 (0-6) | 5 (1-6) | 0 | 0 |  |
|  | Ocrelizumab <3 months from last infusion to vaccination | 2 (7) | 2 (28) | 0 | 0 |  |
|  | Ocrelizumab >3 months from last infusion to vaccination | 5 (18) | 5 (72) | 0 | 0 |  |
|  | Methotrexate** | 6 (21) | 0 | 6 (100) | 0 |  |
|  | None | 15 (54) | 0 | 0 | 15 (100) |  |
|  | data are n (%), unless otherwise indicated |  |  |  |  |  |
|  | (1) reference number WHO: WHO/2019-nCoV/Surveillance_Case_Definition/2020.2 |  |  |  |  |  |
|  | * Severeity of infection was defined as follows: Mild = no hospital admission; Moderate = hospital admisson (no ICU); Severe = ICU admission |  |  |  |  |  |
|  | ** stable dose > 6 months |  |  |  |  |  |
