# Supplementary material for: Immune dynamics in SARS-CoV-2 experienced immunosuppressed rheumatoid arthritis or multiple sclerosis patients vaccinated with mRNA-1273
Source: eLife. 2022 Jul 15;11:e77969. doi: 10.7554/eLife.77969 (PMC9337853; doi:10.7554/eLife.77969)
Supplement: Supplementary file 2. [file elife-77969-supp2.docx]

| **Panel** | **Antibody** | **Clone** | **Fluorochrome** | **Dilution** | **Vendor** | **Cat no** |
| --- | --- | --- | --- | --- | --- | --- |
| Immune-phenotype panel | CD45RA | HI100 | BUV395 | 1:400 | BD | 740298 |
|  | CD8 | RPA-T8 | BUV496 | 1:1000 | BD | 612942 |
|  | CD14 | M5E2 | BUV563 | 1:400 | BD | 741360 |
|  | CD95 | DX2 | BUV615 | 1:100 | BD | 752346 |
|  | TCRgd | 11F2 | BUV661 | 1:400 | BD | 750019 |
|  | CD16 | 3G8 | BUV737 | 1:1000 | BD | 612786 |
|  | CD4 | SK3 | BUV805 | 1:800 | BD | 612887 |
|  | CD71 | M-A712 | BV421 | 1:400 | BD | 562995 |
|  | CD20 | 2H7 | BV480 | 1:400 | BD | 566132 |
|  | CD3 | UCHT1 | BV570-P | 1:200 | Biolegend | 300436 |
|  | CD11c | B-ly6 | BV605 | 1:100 | BD | 563929 |
|  | CD27 | L128 | BV650 | 1:100 | BD | 563228 |
|  | CCR6 | 11A9 | BV711 | 1:100 | BD | 563923 |
|  | HLA-DR | G46-6 | BV750 | 1:800 | BD | 746912 |
|  | CD45RB | MT4 (6B6) | BV786 | 1:400 | BD | 744655 |
|  | CD57 | NK-1 | BB515 | 1:400 | BD | 565285 |
|  | PD1 | EH12.1 | BB700 | 1:400 | BD | 566460 |
|  | CD138 | MI15 | PE | 1:100 | BD | 552026 |
|  | CD10 | HI10a | PE-CF594 | 1:400 | BD | 562396 |
|  | CD56 | B159 | PE-Cy5 | 1:100 | BD | 555517 |
|  | CD24 | ML5 | PE-Cy7 | 1:200 | BD | 561646 |
|  | CD38 | HIT2 | APC | 1:50 | BD | 555462 |
|  | CD19 | SJ25C1 | R718 | 1:400 | BD | 566946 |
|  | CD21 | Bu32 | APC/FIRE750 | 1:400 | Biolegend | 354920 |
|  | BD Brilliant Stain buffer plus | | | | BD | 566385 |
| CD4/CD8 activation panel | CD45RA | HI100 | BUV395 | 1:600 | BD | 740298 |
|  | CD8 | RPA-T8 | BUV496 | 1:1000 | BD | 612942 |
|  | CD27 | L128 | BUV563 | 1:400 | BD | 748705 |
|  | CD38 | HIT2 | BUV615 | 1:200 | BD | 751138 |
|  | CD137 | 4B4-1 | BUV661 | 1:200 | BD | 741642 |
|  | CD40-L | TRAP1 | BUV737 | 1:200 | BD | 748983 |
|  | CD4 | SK3 | BUV805 | 1:800 | BD | 612887 |
|  | CD127 | HIL-7R-M21 | BV421 | 1:200 | BD | 562436 |
|  | CD15S | CSLEX1 | BV510 | 1:800 | BD | 563529 |
|  | CD3 | UCHT1 | BV570-P | 1:200 | Biolegend | 300436 |
|  | CCR7 | 2-L1-A | BV605 | 1:100 | BD | 566754 |
|  | TIGIT | 741182 | BV650 | 1:400 | BD | 747840 |
|  | CXCR3 | 1C6/CXCR3 | BV711 | 1:200 | BD | 563156 |
|  | HLA-DR | G46-6 | BV750 | 1:800 | BD | 746912 |
|  | TIM-3 | 7D3 | BV786 | 1:200 | BD | 742857 |
|  | CCR6 | 11A9 | BB515 | 1:100 | BD | 564479 |
|  | PD1 | EH12.1 | BB700 | 1:400 | BD | 566460 |
|  | ICOS | DX29 | PE | 1:100 | BD | 557802 |
|  | CCR4 | 1G1 | PE-CF594 | 1:400 | BD | 565391 |
|  | CTLA-4 | BNI3 | PE-Cy5 | 1:400 | BD | 555854 |
|  | CD25 | M-A251 | PE-Cy7 | 1:400 | BD | 557741 |
|  | CXCR5 | RF8B2 | APC-R700 | 1:200 | BD | 565191 |
|  | CD226 | 11A8 | APC/FIRE750 | 1:100 | Biolegend | 338320 |
|  | BD Brilliant Stain buffer plus | | | | BD | 566385 |
