# Supplementary material for: Immune dynamics in SARS-CoV-2 experienced immunosuppressed rheumatoid arthritis or multiple sclerosis patients vaccinated with mRNA-1273
Source: eLife. 2022 Jul 15;11:e77969. doi: 10.7554/eLife.77969 (PMC9337853; doi:10.7554/eLife.77969)
Supplement: Transparent reporting form [file elife-77969-transrepform1.docx]

***eLife’s* transparent reporting form**

We encourage authors to provide detailed information *within their submission* to facilitate the interpretation and replication of experiments. Authors can upload supporting documentation to indicate the use of appropriate reporting guidelines for health-related research (see [EQUATOR Network](http://www.equator-network.org/%20)), life science research (see the [BioSharing Information Resource](https://biosharing.org/)), or the [ARRIVE guidelines](http://www.plosbiology.org/article/info:doi/10.1371/journal.pbio.1000412) for reporting work involving animal research. Where applicable, authors should refer to any relevant reporting standards documents in this form.

If you have any questions, please consult our Journal Policies and/or contact us: [editorial@elifesciences.org](mailto:editorial@elifesciences.org).

**Sample-size estimation**

- You should state whether an appropriate sample size was computed when the study was being designed
- You should state the statistical method of sample size computation and any required assumptions
- If no explicit power analysis was used, you should describe how you decided what sample (replicate) size (number) to use

Please outline where this information can be found within the submission (e.g., sections or figure legends), or explain why this information doesn’t apply to your submission:

The sample size was determined by the availability of samples. Information on the sample size and recruitment can be found in

- First paragraph of the Results
- First paragraph of the Materials and Methods
- Figure 1a and b
- Supplemental table 1
- Limitations sections, discussing the limited sample size

**Replicates**

- You should report how often each experiment was performed
- You should include a definition of biological versus technical replication
- The data obtained should be provided and sufficient information should be provided to indicate the number of independent biological and/or technical replicates
- If you encountered any outliers, you should describe how these were handled
- Criteria for exclusion/inclusion of data should be clearly stated
- High-throughput sequence data should be uploaded before submission, with a private link for reviewers provided (these are available from both GEO and ArrayExpress)

Please outline where this information can be found within the submission (e.g., sections or figure legends), or explain why this information doesn’t apply to your submission:

Experiments could not be replicated as deep-immune profiling of cellular immune responses via high-dimensional flow cytometry analysis requires fresh whole blood However, flow cytometry settings were corrected based on the inclusion of rainbow beads for each individual experiment, which allowed us to compare longitudinal samples.

Information on the experimental design can be found in the M&M

Participants were only excluded based on the predefined exclusion criteria described in Fig 1a. No data was excluded based on their responses.

No use of high throughput sequencing

**Statistical reporting**

- Statistical analysis methods should be described and justified
- Raw data should be presented in figures whenever informative to do so (typically when N per group is less than 10)
- For each experiment, you should identify the statistical tests used, exact values of N, definitions of center, methods of multiple test correction, and dispersion and precision measures (e.g., mean, median, SD, SEM, confidence intervals; and, for the major substantive results, a measure of effect size (e.g., Pearson's r, Cohen's d)
- Report exact p-values wherever possible alongside the summary statistics and 95% confidence intervals. These should be reported for all key questions and not only when the p-value is less than 0.05.

Please outline where this information can be found within the submission (e.g., sections or figure legends), or explain why this information doesn’t apply to your submission:

A description of the statistical analyses can be found in the M&M and are described in the respective figure legends.

All figures mention exact p values both significant and not significant, except for the Boolean stack plots for the number of dynamic markers that were expressed. This was a decision made to enhance the readability of the figure for the reader as this would be a lot of non-significant values stacked on top of each other. However, these can be added upon request.

(For large datasets, or papers with a very large number of statistical tests, you may upload a single table file with tests, Ns, etc., with reference to sections in the manuscript.)

**Group allocation**

- Indicate how samples were allocated into experimental groups (in the case of clinical studies, please specify allocation to treatment method); if randomization was used, please also state if restricted randomization was applied
- Indicate if masking was used during group allocation, data collection and/or data analysis

Please outline where this information can be found within the submission (e.g., sections or figure legends), or explain why this information doesn’t apply to your submission:

This is not an experimental study, hence no influence on how experimental groups were established.

**Additional data files (“source data”)**

- We encourage you to upload relevant additional data files, such as numerical data that are represented as a graph in a figure, or as a summary table
- Where provided, these should be in the most useful format, and they can be uploaded as “Source data” files linked to a main figure or table
- Include model definition files including the full list of parameters used
- Include code used for data analysis (e.g., R, MatLab)
- Avoid stating that data files are “available upon request”

Please indicate the figures or tables for which source data files have been provided:

This information will be made available in an online repository upon acceptance/publication of the manuscript.
